# Two-Stage Stochastic Optimization for the Pre-Position and Reconfiguration of Microgrid Defense Resources against Natural Disasters

**DOI:** 10.3390/s22166046

**Published:** 2022-08-12

**Authors:** Bo Jiang, Hongtao Lei, Shengjun Huang, Wenhua Li, Peng Jiao

**Affiliations:** 1 College of Systems Engineering, National University of Defense Technology, Changsha 410073, China; 2 Hunan Key Laboratory of Multi-Energy System Intelligent Interconnection Technology, Changsha 410073, China

**Keywords:** microgrid reconfiguration, stochastic programming, mobile energy storage vehicle, tie line

## Abstract

With the aggravation and evolution of global warming, natural disasters such as hurricanes occur more frequently, posing a great challenge to large-scale power systems. Therefore, the pre-position and reconfiguration of the microgrid defense resources by means of Mobile Energy Storage Vehicles (MEVs) and tie lines in damaged scenarios have attracted more and more attention. This paper proposes a novel two-stage optimization model with the consideration of MEVs and tie lines to minimize the shed loads and the outage duration of loads according to their proportional priorities. In the first stage, tie lines addition and MEVs pre-position are decided prior to a natural disaster; in the second stage, the switches of tie lines and original lines are operated and MEVs are allocated from staging locations to allocation nodes according to the specific damaged scenarios after the natural disaster strikes. The proposed load restoration method exploits the benefits of MEVs and ties lines by microgrid formation to pick up more critical loads. The progressive hedging algorithm is employed to solve the proposed scenario-based two-stage stochastic optimization problem. Finally, the effectiveness and superiority of the proposed model and applied algorithm are validated on an IEEE 33-bus test case.

## 1. Introduction

In recent years, hurricanes, earthquakes, and other natural disasters have caused large-scale power interruptions for a long time, which has brought huge economic losses to society. Forexample, in 2008, China’s extreme snow and ice disaster damaged power grid components in 13 provinces, witha total of 36,740 line faults, 2018 substations affected, andpower grids in some areas collapsed and even entered isolated network operation[1]. Hurricane Sandy in 2012 destroyed the elevated distribution system in New York, resulting in the loss of nearly 1000 power poles and more than 900 transformers, andthe power failure of 8,371,242 customers[2]. It can be seen from the power outage events in recent years that the power production, transmission, anddistribution modes of centralized power generation and long-distance transmission of large units can not fully meet the power demand. Therefore, research on the resilient power grid that can quickly respond to natural disasters has become a trending topic among scholars and has also become an urgent demand for a stable and reliable energy supply in today’s society. Asa kind of Distributed Generation (DG), Mobile Energy Storage Vehicles (MEVs) play a pivotal role in the construction of resilient microgrids. Withthe characteristics of flexibility and stability, MEVs can quickly move to the damaged areas and supply power to the isolated nodes by forming multi-microgrids after a natural disaster occurs, greatly reducing the blackout time. However, due to the high cost of mobile energy storage vehicles, it is uneconomical to use them in large quantities. Inaddition to the installation of mobile energy storage vehicles, another measure to enhance resilience is the addition of tie lines. When some of the original transmission lines are damaged and failed, thetie lines can be closed remotely to restore those isolated island nodes, which costs less than the installation of mobile vehicles. Thus, bythe usage of tie lines and MEVs, when the fault occurs, MEVs and tie lines can be used to reconstruct the power grid to form a local power supply area, so as to realize the rapid recovery of power lossload.

Most literature focuses on the planning of defense resources before power system damage and the allocation of reconfiguration resources and network topology reconfiguration after power system damage. Leietal.[3] proposed a two-stage microgrid reconfiguration model which contained pre-positioning and real-time allocation of mobile generators. Furthermore, different from[3], only critical line switches were considered available in network reconfiguration. In[4], adistributed multiagent coordination scheme of communication network besides the reconfiguration of electric network was proposed for autonomous communication. Gilanietal.[5] considered different types of DGs in microgrid formation and used the time series forecasting method to model the loaduncertainty.

A series of models have been proposed to formulate this issue. In[6], Guoetal. formulated the distribution network reconfiguration as a mixed-integer quadratic programming problem by using electric vehicles and remote-controlled switches to minimize energy loss and switching operation times, withconsidering the battery degradation cost. Maetal.[7] proposed a two-stage stochastic mixed-integer linear program model by taking measures such as line hardening, installing DGs, and adding switches in transmission lines. Ranjbaretal.[8] built a two-stage stochastic planning model by using distributed energy resources and considering the different operation modes in normal and emergency conditions and classified the emergency scenarios into medium, serious and complete damage according to the degree of damage. In[9], Ghasemietal. proposed a three-stage stochastic planning model by hardening lines and placing DGs. They also considered line damage uncertainty compared to the traditional two-stage planning model. Baghbanzadehetal.[10] built a tri-level Defender–Attacker–Defender (DAD) model by using distribution generation to enhance the resilience of distribution networks. Leietal.[11] built a multi-period DAD model to realize the defense resources planning and allocation with the optimization objective of minimizing the shed loads. Mousavizadehetal.[12] firstly defined the index of power system resilience, andthen constructed a two-stage linear model using DGs such as renewable energy which studied the optimal management of energy storage units and DG units and evaluated the resilience of the system. Agrawaetal.[13] proposed a three-stage self-healing method with distributed energy resources to restore maximum priority loads. To sum up, these models can be roughly classified as either deterministic or stochastic. Since the damaged scenario is uncertain, stochastic methods are better suited for the solutions of the microgrid reconfigurationproblem.

Accordingly, some algorithms were designed to solve these problems. Dingetal.[14] used a heuristic algorithm to solve the mixed-integer linear programming problem of power grid reconfiguration, which resulted in multiple solutions with different rules and interests. Huangetal.[15] provided a targeted algorithm based on the nested column-and-constraint generation decomposition to solve a two-stage robust mixed-integer optimization model. Chandaetal.[16] used a two-stage reconfiguration algorithm to enhance the resiliency of the power system after developing a new method to quantify the resiliency which was based on complex network analysis and network percolation theory. In[17], thetraditional distribution system was transformed into multiple autonomous microgrids by optimizing the scale and location of DGs to improve the resiliency of the distribution system, andparticle swarm optimization and genetic algorithm were used to solve the problem. In[18], the key infrastructure nodes in the network were sorted according to their priorities, anda customized PSO algorithm to allocate DGs was designed to maximize resilience and minimize power loss. Khalilietal.[19] proposed a multi-objective optimization model to construct multi-microgrids, which was solved by an exchange marketalgorithm.

With regard to the use of reconfiguration resources, Taherietal.[20] not only considered the pre-positioning and scheduling of mobile energy storage vehicles but also crews who could operate switches. Zhouetal.[21] proposed a distributed fixed-time secondary control scheme that was based on a general directed communication graph. Xuetal.[22] considered the dispatch of repair crews in addition to mobile power sources in the transportation system to restore critical loads. Zhangetal.[23] built a three-stage stochastic planning model for mobile emergency generator allocation which added a capacity decision-making procedure in the first stage compared with the two-stage model. Erenougluetal.[24] proposed a mixed-integer quadratic programming model for dispatching and scheduling multiple types of sources including mobile energy storage systems, mobile emergency generators, and repair crews. Combing through the literature found that mobile energy storage vehicles as a kind of DG were widely studied and applied to the microgrid reconfiguration. However, few studies focused on the application of tie lines, andthey only studied the use of existing tie lines in the power system without considering the addition of tie lines. Shietal.[25] considered the use of tie lines in addition to original line switches for network reconfiguration, andproposed an algorithm that was based on an incidence matrix to identify radial network topology. In[26], tie lines were also utilized in network reconfiguration, andthe master–slave control method was deployed to overcome the challenge of voltage loops.

Based on the above discussion, ascenario-based stochastic two-stage mixed-integer linear program model based on hybrid microgrid defense resources is proposed in this paper. This model includes the pre-position of the MEVs and the location selection of tie line addition before damage, andthe dynamic dispatching of MEVs and operation of added tie lines after damage. Amore detailed problem statement will be presented in Section 2.

The major contributions of this study can be summarized as:

(1) The multi-stage application of tie lines including addition and operation is firstly proposed and studied. This novel method effectively enhances power system resilience and the diversity of reconfiguration resources;
(2) A two-stage framework for the combination use of microgrid defense resources by dispatching MEVs and employing tie lines is firstly proposed, which can restore the critical loads immediately with the limited use of MEVs in severely damaged scenarios;
(3) A scenario-based two-stage mixed-integer linear program model is formulated and explained in detail, which is generalizable and easy to understand.
(4) Several experiments have been conducted to compare the performance of the proposed method with other methods, demonstrating that the combined optimization model with MEVs and tie lines is competitive.

The remainder of this paper is organized as follows. Section 2 describes the action mechanisms of MEVs and tie lines on power grid restoration and proposes a two-stage microgrid reconfiguration framework. Section 3 formulates the two-stage model. In Section 4 the progressive hedging algorithm is discussed, followed by Section 5 which presents numerical case studies. Finally, thepaper is concluded in Section 6.

## 2. ProblemStatement

In this section, we first depict how natural disasters damage the power grid. Based on this, theaction mechanisms of MEVs and tie lines on power grid restoration are described, anda two-stage microgrid reconfiguration framework of combination use of MEVs and tie lines is proposed andillustrated.

### 2.1. Mechanism of MEVs on Power GridRestoration

**Figure 1:**
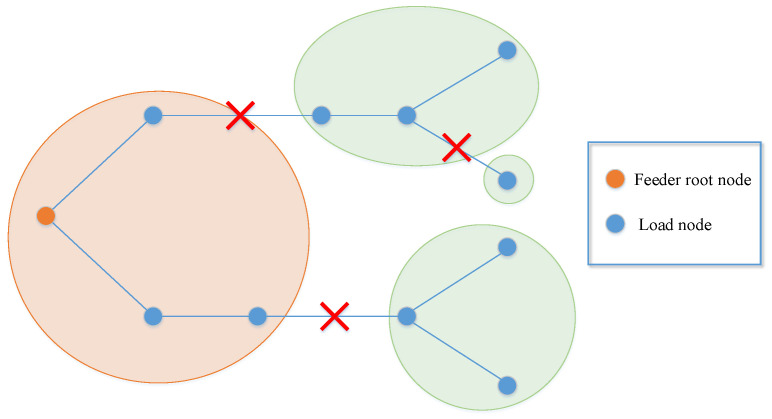
The attacked network topology.

**Figure 2:**
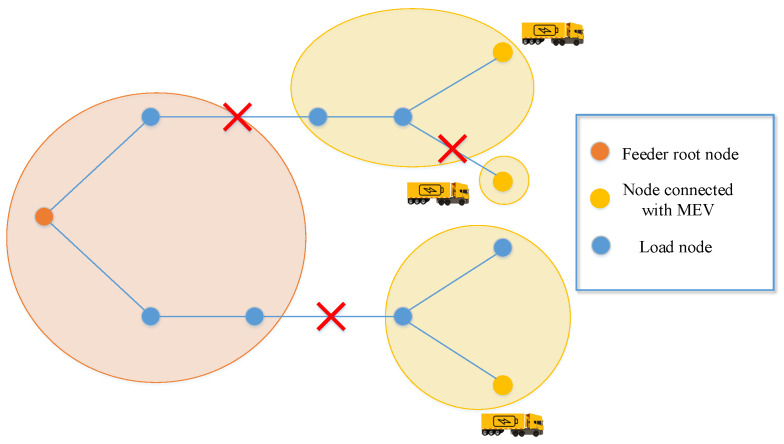
Network reconfiguration with MEVs.

Natural disasters and man-made attacks can usually destroy parts of lines in the power system, causing some nodes to lose connection with the main network and become isolated island loads. Asshown in Figure 1, afterthe attack from the outside world, thedistribution system is split into four areas, ofwhich the orange area is the normal power supply area connected with the feeder root node, andthe three green areas are island loads. Thetraditional restoration method only changes the topology of the power system by operating the line or node switches, which may not completely restore the island loads. Moreover, it takes a lot of time to repair the damaged lines manually, which will cause huge losses to critical nodes that do not have backup power. DG is a promising new energy utilization tool of microgrid, which has the advantages of low environmental pollution and flexible control means. Inall kinds of DGs, MEVs can move between different load nodes, andprovide load supply, voltage regulation, and other services. When users cannot fully access the main power grid, MEVs are the key resources that can quickly restore power supply for some important loads and realize the rapid restoration of power services across the power system. Especially after the microgrid is attacked, MEVs combined with transmission line switches can be used to form multi-microgrids to restore power supply for important loads, shortening the outage time and reducing the outage scope. Asshown in Figure 2, the3 green areas turn into yellow due to the use of MEVs, andthe nodes connected with MEVs become power nodes to supply power for themselves and other load nodes.

### 2.2. Mechanism of Tie Lines on Power Grid Restoration

**Figure 3:**
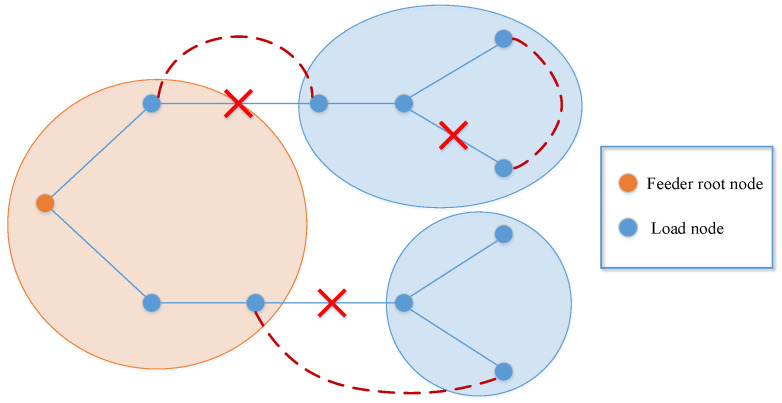
Network reconfiguration with tie lines.

As shown in Figure 3, the three red dashed lines represent the added tie lines, of which the status is closed. Therefore, the three green areas representing island loads in Figure 1 turn into two blue areas which represent the island loads that are restored. It is obvious that the use of tie lines can quickly change the topology of the power network and make the unserved loads connect to the main network to get supplied immediately. In this work, we add limited tie lines (default to open) between some nodes in the first stage and decide whether to close the switches of them in the second stage by optimizing the proposed model. Thus, once the original transmission lines are damaged, the tie lines as backup lines can be closed in very little time to change the topology to restore loads.

### 2.3. A Two-Stage Framework: Combination Use of MEVs and Tie Lines

**Figure 4:**
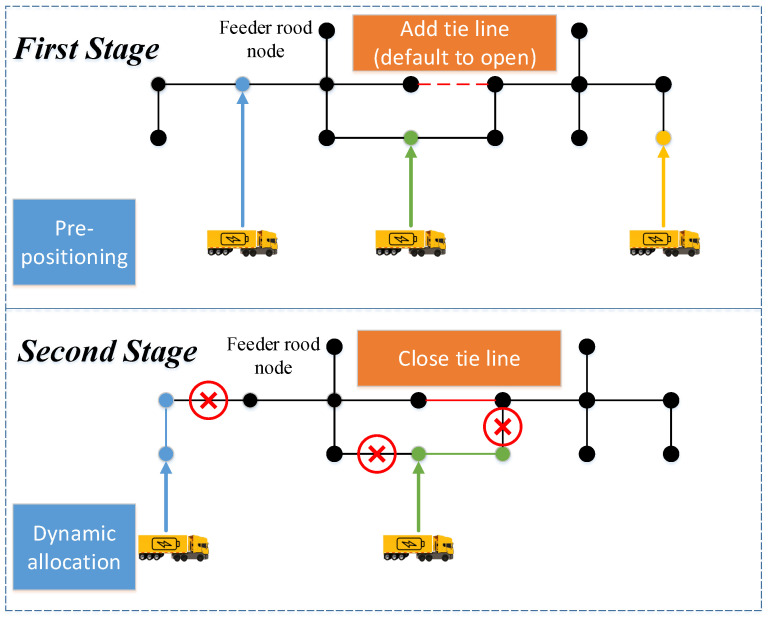
The illustration of the two-stage framework.

In the new mode of microgrid reconfiguration with the combination use of MEVs and tie lines, after the original line faults occur, several local power supply areas can be formed by the deployment of MEVs and tie lines more flexibly, so as to realize the rapid restoration of unsupplied loads. As shown in Figure 4, in the first stage (pre-positioning stage), limited MEVs are pre-positioned to some staging location nodes and limited tie lines are added between some node pairs. In the second stage (dynamic allocation stage), each MEV will be sent to the allocation node. Each tie line’s status will be decided to be kept open or closed. We assume that the hardened tie lines will not be damaged in the second stage. Consequently, it is a two-stage microgrid reconfiguration problem considering MEVs dispatching and tie lines usage. Compared with the traditional reconfiguration method, the model proposed in this paper is more complex than those which only consider the use of DGs or topology reconfiguration by changing the status of line switches. Due to the use of tie lines, the radial topology constraint is also an important issue to be figured out.

## 3. Problem Formulation

In this section, the model formulation for the proposed problem is proposed.

### 3.1. Pre-Positioning Stage

#### 3.1.1. Objective Function


(1) $$\begin{array}{c} \min\sum_{n\in N}w_{n}\left\{\sum_{i\in B}\alpha_{i}p_{i}\left[a_{1}\sum_{k\in D}\sum_{s\in S}\sum_{m\in M}v_{mskn}t_{skn}+a_{2}\sum_{i\in B}sl_{in}\right]\right\} \end{array}$$

The objective (1) is to minimize the expected outage time of loads and the amount of loads to be shed, considering the weight of each scenario. The two goals are expressed as the first and second terms in square brackets, respectively. The energy demand of the loads and the corresponding weight are indicated outside the square brackets. Specifically, the first term $\sum_{k\in D}\sum_{s\in S}\sum_{m\in M}v_{mskn}t_{skn}$ refers to the time required to recover nodes by using MEVs. Since tie lines are deployed before natural disasters, the time of operating the switches of tie lies is negligible. Considering adding tie lies and dispatching MEVs require high investment costs, one of the goals of this stage is to make the average shed loads as small as possible when limited reconfiguration resources are used. Thus, the second term ${sl}_{in}$ is the shed loads on node *i* under scenario *n*. Overall, the optimization objective of this problem is to restore more loads in the shortest possible time. $a_{1}$ and $a_{2}$ represent the weight of the two objectives which are decided by decision-makers.

#### 3.1.2. Constraints

The defined objective is subject to the following constraints which are divided into several groups as follows:

Constraints (2)–(4) referring to [3] represent the pre-positioning constraints of MEVs. Constraints (2) maintain that the total number of MEVs pre-positioned in the staging location nodes is no more than their maximum capacity. Constraints (3) ensure that every single MEV can only be pre-positioned to one staging location node. Constraints (4) ensure that the number of MEVs pre-positioned in the first stage can not exceed their budgets.

(2) $$\sum_{m\in M}u_{ms}\leq A{}_{s},\forall s$$

(3) $$\sum_{s\in S}u_{ms}=1,\forall m$$

(4) $$\sum_{m\in M,s\in S}u_{ms}\leq M{}_{n}$$

Constraints (5) and (6) referring to [3] represent the dynamic allocation constraints of MEVs. After each MEV is pre-positioned in a specific staging location node in the first stage, constraints (5) declare that only if there is any MEV *m* in a staging location node, the MEV *m* can be allocated to one of the allocation nodes in each scenario in the second stage. Constraints (6) ensure that every single MEV can only be allocated to one allocation node *k* from the staging location node *s*.

(5) $$\sum_{k\in D}v_{mskn}\leq u_{ms},\forall s,\forall m,\forall n$$

(6) $$\sum_{s\in S}\sum_{m\in M}v_{mskn}\leq 1,\forall k,\forall n$$

Constraints (7)–(11) represent the topology reconfiguration constraints that guarantee the topology of the power system keeps radial all the time. Constraints (7) mean that if there is any MEV allocated to candidate node *k*, then the node *k* is a power node supplied by MEVs. It is noted that the candidate locations include the staging locations and allocation locations where the MEVs can connect to the nodes on them. Constraints (8) mean that if the node *k* is a feeder root node, then the node has a power source. Constraints (9) and (10) mean that the numbers of damaged lines and tie lines must be less than their budgets. Based on graph theory, if the graph is radial, the following two conditions must be satisfied at the same time: (a) the number of closed branches equals the number of buses minus the number of sub-graphs; (b) the connectivity of each sub-graph is ensured. Thus, the number of closed branches including closed original transmission lines and tie lines equals the number of buses minus the number of sub-graphs in constraints (11). We use a depth-first traversal algorithm to find the connected components of an undirected graph in this paper. After traversing from one vertex denoted as node1, all vertices that can be reached through the graph adjacency will be accessed from this vertex. Starting from one of the unreachable vertices, another connected component can be found. Repeating this process until all vertices are accessed, all connected components will be identified. Based on depth-first-search algorithm framework, the specific flowchart of the algorithm to find the sub-graphs of an undirected graph is shown in Algorithm 1.

(7) $$wa{}_{kn}=\sum_{s\in S}\sum_{m\in M}v_{mskn},\forall k\in D,\forall n$$

(8) $$wb{}_{kn}=1,\forall k\in C,\forall n$$

(9) $$\sum_{i,j\in B}{AL}_{ijn}\leq BA,\forall i,\forall n$$

(10) $$\sum_{i,j\in B}TL_{ijn}\leq BT,\forall i,\forall n$$

(11) $$\sum_{i,j\in B}y_{ijn}+\sum_{i,j\in B}STL_{ijn}=B{}_{n}-G{}_{n},\forall i,\forall k,\forall n$$

**Algorithm 1:** Algorithm For Finding Sub-graphs.

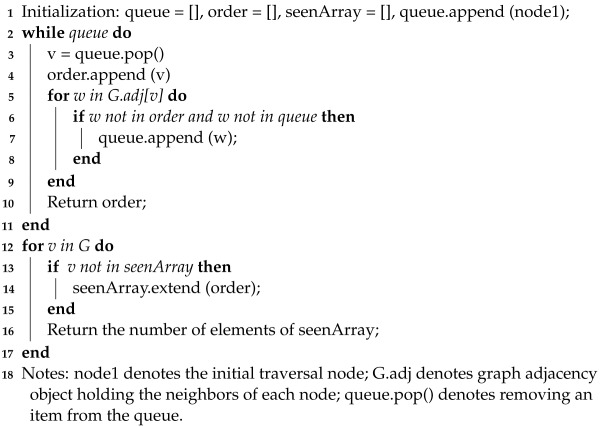



Constraints (12)–(16) represent the power balance constraints. Constraint (12) referring to [11] ensures the inflow and outflow on node *i* are equal to realize power balance. Constraint (13) determines the power flows according to the node phase angles, with binary variables representing line status. If there is a closed transmission line that is not damaged or a tie line between nodes *i* and *j*, then $\left(y_{ijn}+{STL}_{ijn}\right)\left(1-{AL}_{ijn}\right)$ is 1, otherwise 0. Note that there is a nonlinear term on the right side of the equation. For linearization, we introduce the continuous variable $\lambda_{ijn}$ to replace $\left(y_{ijn}+STL_{ijn}\right)\left[\delta_{o(l_{ij})}-\delta_{d(l_{ij})}\right]$, so this constraint can be transformed into the following constraints (17)–(20) equivalently by using the big-M approach. Constraint (14) guarantees that the shed loads on node *i* is between 0 and the demand at the node. Constraints (15) and (16) keep the power supply on node *i* as nonnegative and do not exceed its capacity if there is an MEV or if it is a feeder root node.

(12) $$\begin{array}{c} ga_{in}+gb_{in}-\sum_{l_{ij}\left|o(l_{ij})=i\right.}p_{l_{ij}}+\sum_{l_{ij}\left|d(l_{ij})=i\right.}p_{l_{ij}}+sl_{in}-p_{i}=0,\forall i,\forall n \end{array}$$

(13) $$p{}_{l_{ij}}r{}_{l_{ij}}=(y{}_{ijn}+STL{}_{ijn})(1-AL{}_{ijn})\left[\delta {}_{o(l_{ij})}-\delta {}_{d(l_{ij})}\right],\forall i,\forall n$$

(14) $$0\leq sl{}_{in}\leq p{}_{i},\forall i,\forall n$$

(15) $$0\leq ga{}_{in}\leq wa{}_{kn}Ga,\forall i,\forall n$$

(16) $$0\leq gb{}_{in}\leq wb{}_{kn}Gb,\forall i,\forall n$$

(17) $$\begin{array}{c} -M\left[\left(1-\left(y_{ijn}+STL_{ijn}\right)\right)\right]\leq \lambda_{ijn}-\left[\delta_{o(l_{ij})}-\delta_{d(l_{ij})}\right]\leq M\left[\left(1-\left(y_{ijn}+STL_{ijn}\right)\right)\right] \end{array}$$

(18) $$-M\left(y_{ijn}+STL_{ijn}\right)\leq \lambda_{ijn}\leq M\left(y_{ijn}+STL_{ijn}\right)$$

(19) $$-MAL_{ijn}\leq p_{l_{ij}}r_{l_{ij}}-\lambda_{ijn}\leq MAL_{ijn}$$

(20) $$-M\left(1-AL_{ijn}\right)\leq p_{l_{ij}}r_{l_{ij}}\leq M\left(1-AL_{ijn}\right)$$

Constraints (21)–(24) represent the line status constraints. Among them, constraints (21) and (22) are self-explanatory. Constraints (23) depict that the tie line should not be added if there is an original transmission line between the node pair (*i*, *j*). Constraints (24) describe that the switches of tie lines may be closed only if there exists any tie line.

(21) $$y{}_{ijn}=0,\forall l{}_{ij}\in DL,\forall n$$

(22) $$AL{}_{ijn}=1,\forall l{}_{ij}\in DL,\forall n$$

(23) $$TL{}_{ijn}=0,(i,j)\in L,\forall i,\forall j,\forall n$$

(24) $$STL{}_{ijn}\leq TL{}_{ijn},\forall i,\forall j,\forall n$$

### 3.2. Real-Time Allocation Stage


(25) $$\begin{array}{c} \min\left\{\sum_{i\in B}\alpha_{i}p_{i}\left[a_{1}\sum_{k\in D}\sum_{s\in S}\sum_{m\in M}v_{mskn}t_{skn}+a_{2}\sum_{i\in B}sl_{in}\right]\right\} \end{array}$$

s.t.(2)–(14)

In the first stage, we have determined the optimal pre-positioning decisions of MEVs $u_{ms}$ and the decisions of adding tie lines ${TL}_{ijn}$. In the second stage, according to the damaged lines detected after natural disasters, the dynamic allocation of MEVs from staging locations will be carried out, and the status of tie lines will be decided. The optimization goal of this stage is to minimize the weighted time of load recovery and load shedding when scenario *n* occurs.

## 4. Progressive Hedging Algorithm

The proposed two-stage microgrid optimization problem is where some of the decision variables are binary variables and the data is uncertain is a multi-stage mixed-integer stochastic programming problem, which is difficult to solve in a short time. To represent the uncertainty in data, we use the common approach by formulating a finite number of scenarios with corresponding probabilities for the values of uncertain parameters in this paper. Since the Progressive Hedging Algorithm (PHA) has proved an effective method for solving multi-stage stochastic programming problems [27,28], especially those with discrete decision variables in every stage [29], we apply this algorithm to solve this scenario-based two-stage stochastic optimization problem. Based on [3], the implementation of the PHA is described in Algorithm 2. The PHA decomposes the extended form according to the scenario and iteratively solves the penalized versions of the sub-problems to gradually enhance the realizability, thus reducing the computational difficulty related to large problem instances.
**Algorithm 2:** Progressive Hedging Algorithm.
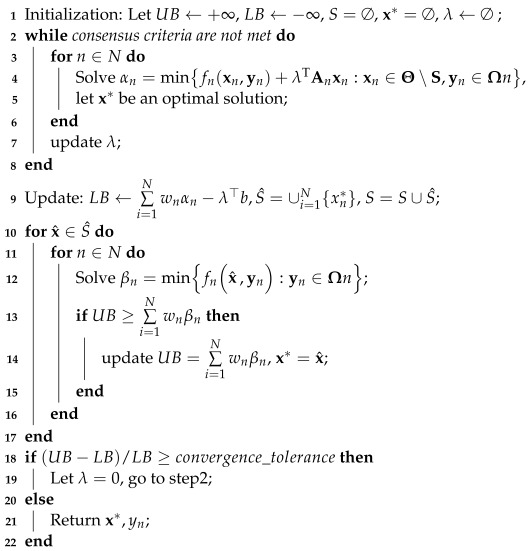


We consider the stochastic program of the following form:

(26) $$\min\left\{\underset{i=1}{\sum^{N}}\;f_{n}\left(x,y_{n}\right):x\in \Theta \;,y_{n}\in \Omega_{n}\right\}\;$$

where x, $y_{n}$, $f_{n}$ denote first-stage decision variables, second-stage decision variables, and second-stage objective function, respectively. Θ is the feasible set of x defined by constraints such as (2), (3) and (10), $\Omega_{n}$ is the feasible set of $y_{n}$ defined by other constraints. Since the first-stage decisions are independent of specific scenarios, we introduce the non-anticipativity constraints to make use of the block-diagonal structure of the problem. Therefore, the scenario-based two-stage stochastic optimization problem can be reformulated as:

(27) $$\min\left\{\underset{i=1}{\sum^{N}}\;f_{n}\left(x_{n},y_{n}\right):x_{n}\in \Theta ,y_{n}\in \Omega_{n},\underset{i=1}{\sum^{N}}\;A_{n}x_{n}=b\right\}\;$$

where the equation $\sum_{i=1}^{N}\;A_{n}x_{n}=b$ is the non-anticipativity constraint enforcing $x_{1}=x_{2}=\ldots =x_{n}$. By dualizing these non-anticipativity constraints, the form of the Lagrangian duality problem is as follows:

(28) $$\begin{array}{c} \max_{\lambda }\;\left.\{LB\left(\lambda \right):=\underset{i=1}{\sum^{N}}\;w_{n}\min\left\{f_{n}\left(x_{n},y_{n}\right)+\lambda^{T}A_{n}x_{n}:x\in \Theta ,y_{n}\in \Omega_{n}\right\}-\lambda^{⊤}b\right.\}\; \end{array}$$

where λ is the dual vector; $w_{n}$ is the weight of scenario *n*. As for the consensus criteria in step2, it is based on the average per-scenario deviation from the average denoted as pd:

(29) $$pd=\sum_{l,n}\frac{|x_{n}\left(l\right)-{\bar{x}}_{n}\left(l\right)|}{N}\leq \underline{pd}\;$$

where $\underline{pd}$ denotes a threshold set as 0.1 in this paper; ${\bar{x}}_{n}$ denotes the weighted average of $x_{n}$ in the previous iteration; *l* denotes the *l*th element of the corresponding vector; **N** denotes the number of scenarios.

## 5. Numerical Cases and Analyses

### 5.1. Experimental Setup

**Figure 5:**
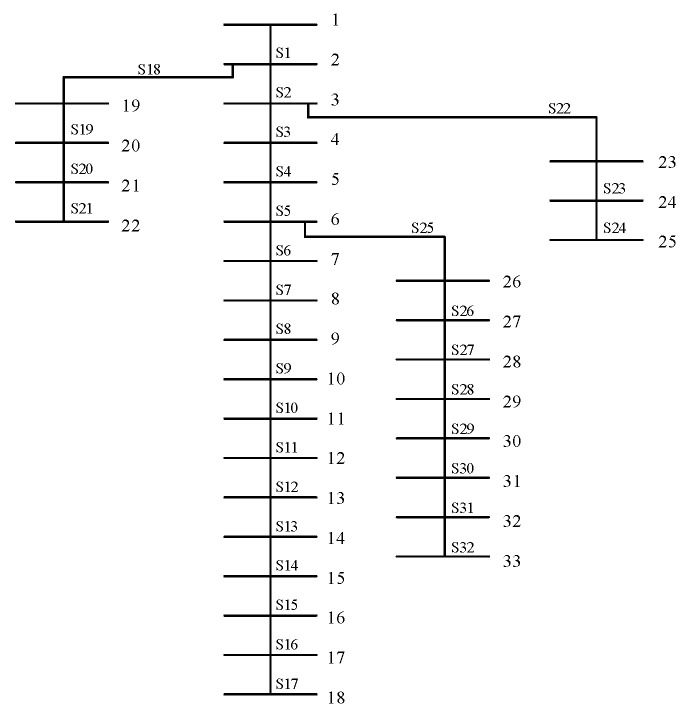
A modified IEEE 33-bus test system.

In this section, case studies are performed in the IEEE 33-node distribution system to validate the effectiveness and superiority of the two-stage 0-1 stochastic programming problem. As in [26], the topology of this system is presented in Figure 5. This distribution system consists of 33 nodes and 32 edges, where node 1 is a generation node and each edge has a remote control switch on it. As illustrated in Figure 5, the switch of the edge between nodes 1 and 2 is denoted as S1. The parameters of nodes and branches such as resistance and reactance can be found in [30]. The computational tasks are performed on a personal computer with an Intel Core i7 Processor (2.60 GHz) and 64-GB RAM, and the code is implemented via Gurobi 9.0.3 with the default setting.

### 5.2. Demonstration of the Proposed Two-Stage Optimization Framework

Due to the lack of actual damaged data, this paper uses the normal distribution to randomly generate 10 random damaged scenarios and load priorities (weights are set to 5, 3, 1). Considering the influence of vehicle transport speed in different damaged scenarios, this paper generates vehicle transport speed in the speed set {60 Km/h, 90 Km/h, 100 Km/h} with equal probability randomly. The maximum power of MEV is set as 1000 kW. When setting the parameters of the routing network, we first generate the adjacency matrix between nodes according to the damaged scenario and then calculate the transportation time of each vehicle on the corresponding routing according to the given speed parameters. In order to compare the effects of different reconfiguration strategies on the optimization objectives, multiple comparative experiments are carried out.

**Figure 6:**
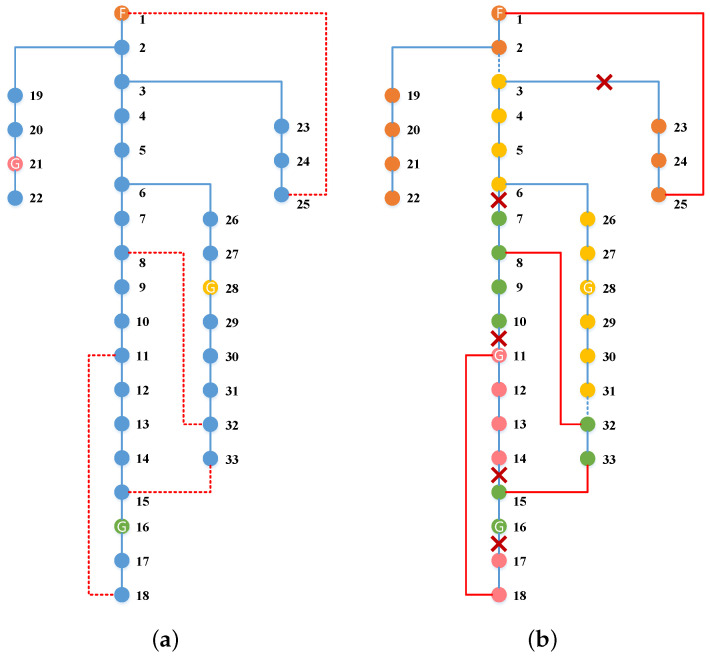
Microgird reconfiguration with MEVs and tie lines for 33-bus system. (**a**) The first stage. (**b**) The second stage.

As shown in Figure 6, we select a typical scenario to illustrate the proposed two-stage reconfiguration solution. In Figure 6, the blue and red lines represent original transmission lines and tie lines, respectively. The dotted lines and solid lines represent the open and close status of line switches, respectively. The orange node marked F is the feeder root node, and the yellow, green, and pink nodes marked G are the corresponding nodes connected with MEVs 1, 2, and 3, respectively. It can be observed in Figure 6a that the MEVs are firstly pre-positioned to staging locations of nodes 28, 16, and 21, and tie lines are added between node pairs of $\left\{(1,25),(8,32),(11,18),(15,33)\right\}$ in the first stage.

After the extreme situation, the faulted lines in the real scenario are detected which are marked with a cross in Figure 6b. Then MEVs are sent from staging locations to allocation locations, and the switches of tie lines and original lines are operated to be close or open to form microgrids to restore critical loads. The first and second stage decisions for the demonstration case are shown in Table 1 and Table 2. In the second stage, MEVs 1 and 2 continue to stay in their staging locations, while MEV 3 is sent to node 11 from node 21. In addition, the switches of four added tie lines are operated to close and the switches of the original lines (2, 3) and (31, 32) are operated to open, while other switches keep closed to maintain the radial structure of the power system. As shown in Figure 6b, the power system is divided into four microgrids power by nodes 1, 28, 11, and 16, respectively. For the sake of intuitiveness, the nodes in different microgrids are represented in different colors which are corresponding to the power nodes. For instance, the nodes of $\left\{7,8,9,10,15,32,33\right\}$ turn green since they are supplied by node 16, which is connected with MEV 2.

### 5.3. Comparisons with Other Methods

In order to further reflect the superiority of our proposed model, we compare it with other methods which only consider using MEVs or tie lines. The models of these two methods are easy to build according to the proposed two-stage optimization framework.

#### 5.3.1. Model of Only Using MEVs

The objectives of the two stages are the same as objectives (1) and (25). The constraints are a little bit different from the proposed model, which are as follows.

s.t.(2)–(9), (14)–(22)

(30) $$\sum_{i,j\in B}y_{ijn}=B_{n}-G_{n},\forall i,\forall k,\forall n$$

(31) $$p_{l_{ij}}r_{l_{ij}}=y_{ijn}\left(1-AL_{ijn}\right)\left[\delta_{o(l_{ij})}-\delta_{d(l_{ij})}\right],\forall i,\forall n$$

**Figure 7:**
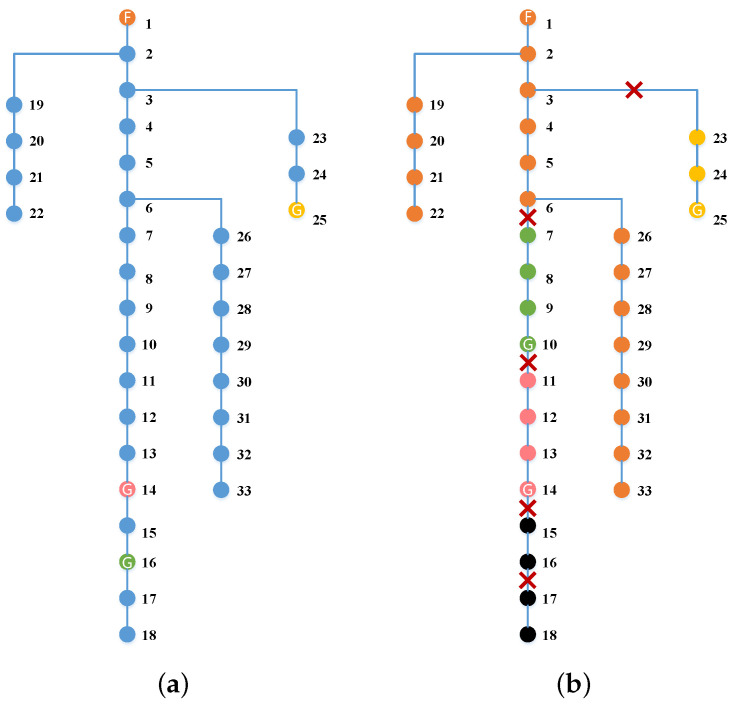
Microgird reconfiguration with MEVs for 33-bus system. (**a**) The first stage. (**b**) The second stage.

The optimization results of this model are shown in Figure 7. Based on the same damaged scenarios, MEVs 1, 2, and 3 are pre-positioned in nodes 25, 16, and 14 in the first stage, respectively. In the second stage, MEVs 1 and 3 continue to stay in their staging locations, while MEV 2 is sent to node 14 from node 16. Thus, four microgrids are formed to restore the critical loads. However, due to the budget limitation of MEVs, this method results in two load islands that are not supplied and represented as black dots, i.e., (15–16), (17–18).

#### 5.3.2. Model of Only Using Tie lines

Since the model of only using tie lines does not include MEVs, the time of dispatching MEVs of objectives (1) and (25) is supposed not to be considered. Its objectives in the first and second stages and constraints that should be satisfied are as follows.

(32) $$\begin{array}{c} \min\sum_{n\in N}w_{n}\sum_{i\in B}sl_{in} \end{array}$$

(33) $$\begin{array}{c} \min\sum_{i\in B}sl_{in} \end{array}$$

s.t.(8)–(14), (16)–(24)

**Figure 8:**
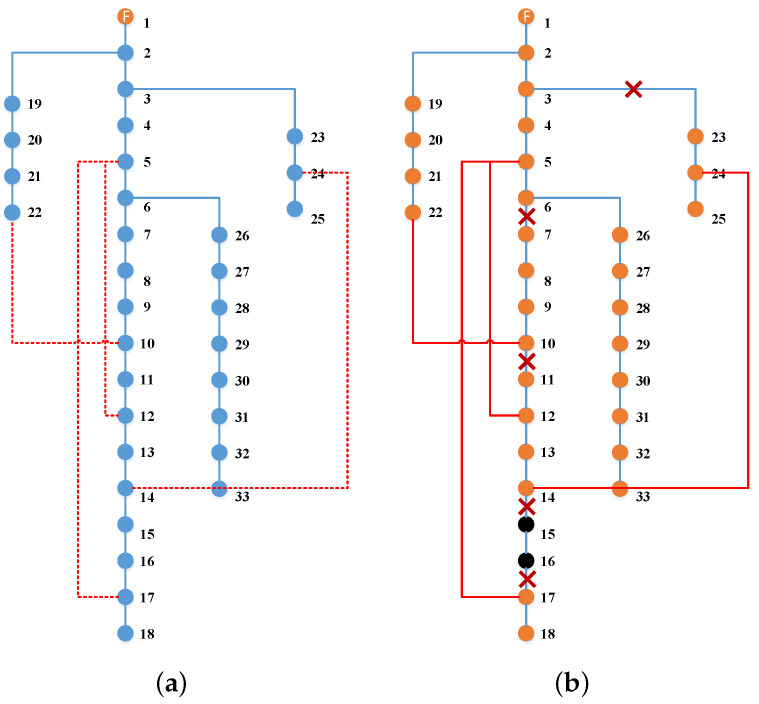
Microgird reconfiguration with tie lines for 33-bus system. (**a**) The first stage. (**b**) The second stage.

The optimization results of this model are shown in Figure 8. In the first stage, the four tie lines are added at node pairs of $\left\{(5,12),(5,17),(10,22),(14,24)\right\}$. After the attack of natural disasters, there remain 5 load islands that are disconnected to the main grid, i.e., (7–8–9–10), (11–12–13–14), (15–16), (17–18), and (23–24–25). In the second stage, the switches of the four tie lines are operated to close, resulting in the load islands, except for (15–16), all being restored. For example, the load island ((7–8–9–10) is restored due to the tie line (10, 22) which makes it connect to the main power grid supplied by the feeder root node. However, due to the budgets of tie lines, there still remain load islands (15–16) that are not supplied.

Overall, both the methods are advantageous to load restoration. However, only using the single configuration resource with the same budgets cannot fully restore all the disconnected loads. The combined use of these two resources can play a greater role in power grid restoration.

### 5.4. Effect of Budgets of Reconfiguration Resources on Load Restoration

In this section, the budgets of MEVs and tie lines are changed to investigate their effects of them on the final reconfiguration results. We use load shedding to measure the effects, which is obtained from the average results of 20 repeated experiments and calculated by Equation (34). As seen in Table 3, when the budgets of MEVs are fixed, the load shedding is decreasing as the budgets of tie lines increase. When the budgets of tie lines are fixed, the load shedding is decreasing as the budgets of MEVs increase.

(34) $$\begin{array}{c} \mathrm{Load}\;\mathrm{Shedding}\;=\;\frac{\sum_{i\in B}\alpha_{i}sl_{in}}{\sum_{i\in B}\alpha_{i}p_{in}},\forall i,\forall n \end{array}$$

## 6. Conclusions

To rapidly recover a microgrid under damaged scenarios, this paper proposes a novel two-stage stochastic mixed-integer model based on the combined optimization of MEVs and tie lines, minimizing the load shedding and the outage duration of loads according to their priorities. Numerical studies on the IEEE 33-bus distribution system demonstrate the effectiveness of the proposed model on restoring critical loads in the microgrid reconfiguration. Compared with the methods which only dispatch MEVs or use tie lines, the combined optimization model can restore more loads at less cost. Moreover, the microgrid recovery effect of the proposed approach improves with the increase of the budgets of reconfiguration resources. In future work, we will consider other reconfiguration resources, such as repair crews and more complex damaged scenarios after multiple extreme events, and design more computationally efficient algorithms.

## Figures and Tables

**Table 1:** MEV Dispatch Scheme.

MEV	Staging Locations		Allocation Locations
1	node 28	→	node 28
2	node 16	→	node 16
3	node 21	→	node 11

**Table 2:** Tie Lines Addition and Operation Scheme.

Tine Line	First Satge		Second Satge
1	add tie lines (1, 25)	→	close the switch
2	add tie lines (8, 32)	→	close the switch
3	add tie lines (11, 18)	→	close the switch
4	add tie lines (15, 33)	→	close the switch

**Table 3:** Load Shedding Under Different Microgrid Defense Resource budgets.

Maximum Damaged Lines	Budgets of MEVs	Budgets of Tie Lines	Load Shedding
9	3	1	8.69%
	3	2	6.38%
	3	3	4.88%
	3	4	2.85%
10	2	5	9.23%
	3	5	6.87%
	4	5	4.36%
	5	5	2.71%

## Data Availability

Not applicable.

## Abbreviations

*Indices and Sets*	
*B*	Set of microgird nodes indexed by *i*.
C,D	Set of feeder root nodes and candidate nodes for MEVs connection indexed by *k*.
*M*	Set of MEVs indexed by *m*.
*S*	Set of staging locations for pre-positioning indexed by *s*.
*L*	Set of transmission lines, $l_{ij}\in L$.
DL	Set of damaged lines, $l_{ij}\in DL$.
*N*	Set of scenarios indexed by *n*.
*Parameters*	
$A_{s}$	Maximum number of MEVs prepared at the staging location *s*.
$B_{n}$	Number of microgird nodes.
$G_{n}$	Number of sub-graphs.
$M_{n}$	Budget of MEVs.
BA	Budget of attacks on transmission lines.
BT	Budget of tie lines.
$G_{a}$	Capacity of an MEV.
$G_{b}$	Capacity of feeder nodes.
$t_{skn}$	Travel time of an MEV from staging location *s* to node *k* under scenario *n*.
$r_{l_{ij}}$	Reactance of transmission line $l_{ij}$.
$o(l_{ij})$	Origin node of transmission line $l_{ij}$.
$d(l_{ij})$	Destination node of transmission line $l_{ij}$.
$w_{n}$	Weight of scenario *n*.
$P_{i}$	Power demand at node *i*.
$P_{l_{ij}}$	Power flow of transmission line $l_{ij}$.
*Variables*	
${ga}_{in}$	Generation level of node *i* supported by the candidate nodes under scenario *n*.
${gb}_{in}$	Generation level of node *i* supported by the feeder nodes under scenario *n*.
${sl}_{in}$	Shed loads on node *i* under scenario *n*.
$l_{ij}$	Binary, 1 if there is a transmission line connecting node *i* and node *j*, 0 otherwise.
${TL}_{ijn}$	Binary, 1 if there is a tie line(defaults to open) connecting node *i* and node *j* under scenario *n*, 0 otherwise.
${STL}_{ijn}$	Binary, 1 if the switch of the tie line ${TL}_{ijn}$ is closed, 0 otherwise.
${AL}_{ijn}$	Binary, 1 if line (i,j) is attacked under scenario *n*, 0 otherwise.
$u_{ms}$	Binary, 1 if MEV *m* is pre-positioned to staging location *s*, 0 otherwise.
$v_{mskn}$	Binary, 1 if MEV *m* is real-time allocated (sent) from staging location *s* to node *k* under scenario *n*, 0 otherwise.
${wa}_{kn}$	Binary, 1 if an MEV is connected to node *k* under scenario *n*, 0 otherwise.
${wb}_{kn}$	Binary, 1 if node *k* is a feeder root node under scenario *n*, 0 otherwise.
$y_{ijn}$	Binary, 1 if line (i,j) is closed under scenario *n*, 0 otherwise.
λ	The auxiliary binary variable to replace the nonlinear term $\left(y_{ijn}+STL_{ijn}\right)\left[\delta_{o(l_{ij})}-\delta_{d(l_{ij})}\right]$.
